# Supplementary material for: Broad-Spectrum In Vitro Activity of Nα-Aroyl-N-Aryl-Phenylalanine Amides against Non-Tuberculous Mycobacteria and Comparative Analysis of RNA Polymerases
Source: Antibiotics (Basel). 2024 Apr 28;13(5):404. doi: 10.3390/antibiotics13050404 (PMC11117372; doi:10.3390/antibiotics13050404)
Supplement: Supplementary file 1 [file antibiotics-13-00404-s001.zip › antibiotics-2975541-supplementary.pdf]

# Supplementary Information

## Broad-Spectrum *In Vitro* Activity of *N*α-aroyl-*N*-aryl-Phenylalanine Amides Against Non-Tuberculous Mycobacteria and Comparative Analysis of RNA Polymerases

***Table S 1.*** *$MIC_{90}$ values of a selection of AAPs against type strains of the Mycobacterium abscessus complex. Cell shading from green to red indicates high to low activity. The displayed values are average values of two technical replicates. For detailed information on the origin of clinical isolates see Materials & Methods section of the main manuscript.*

| | *M. abscessus* subsp. ***abscessus*** ATCC 19977 | *M. abscessus* subsp. ***massiliense*** CCUG 48898-T | *M. abscessus* subsp. ***bolletii*** CCUG 50184-T |
|---|---|---|---|
| ID | $MIC_{90}$ [µM] | $MIC_{90}$ [µM] | $MIC_{90}$ [µM] |
| **CLR** | 1.5 | 0.2 | 0.5 |
| **MMV** | 6.4 | 9.1 | 4.4 |
| **1** | 7.3 | 5.7 | 1.9 |
| **2** | 4.7 | 4.8 | 2.1 |
| **3** | 2.1 | 2.8 | 8.5 |
| **4** | 2.6 | 4.4 | 3.6 |
| **5** | 1.5 | 2.3 | 2.3 |
| **6** | 5.0 | 4.0 | 2.0 |
| **7** | 1.8 | 2.0 | 2.1 |
| **8** | 1.5 | 1.4 | 1.7 |
| **9** | 4.8 | 4.4 | 6.5 |
| **10** | 2.2 | 2.6 | 9.0 |

***Table S 2.*** *$MIC_{90}$ values of a selection of AAPs against a panel of Mycobacterium abscessus complex clinical isolates. Cell shading from green to red indicates high to low activity. The displayed values are average values of two technical replicates. For detailed information on the origin of clinical isolates see Materials & Methods section of the main manuscript.*

| | *M. abscessus* subsp. ***abscessus*** Bamboo | *M. abscessus* subsp. ***abscessus*** M9 | *M. abscessus* subsp. ***abscessus*** M199 | *M. abscessus* subsp. ***abscessus*** M337 | *M. abscessus* subsp. ***abscessus*** M404 |
|---|---|---|---|---|---|
| ID | $MIC_{90}$ [µM] | $MIC_{90}$ [µM] | $MIC_{90}$ [µM] | $MIC_{90}$ [µM] | $MIC_{90}$ [µM] |
| **CLR** | 0.5 | 6.7 | 19.5 | 7.3 | 0.8 |
| **MMV** | 8.2 | 26.4 | 29.3 | 9.8 | 14.2 |
| **7** | 2.1 | 8.1 | 9.5 | 3.5 | 6.1 |
| **8** | 2.3 | 5.8 | 9.3 | 3.9 | 7.0 |

| | *M. abscessus* subsp. ***abscessus*** M422 | *M. abscessus* subsp. ***bolletii*** M232 | *M. abscessus* subsp. ***bolletii*** M506 | *M. abscessus* subsp. ***massiliense*** M111 |
|---|---|---|---|---|
| ID | $MIC_{90}$ [µM] | $MIC_{90}$ [µM] | $MIC_{90}$ [µM] | $MIC_{90}$ [µM] |
| **CLR** | 4.2 | 10.6 | 1.1 | 0.5 |
| **MMV** | 13.4 | 6.0 | 10.8 | 6.8 |
| **7** | 1.8 | 3.9 | 2.9 | 3.4 |
| **8** | 1.8 | 4.6 | 2.9 | 5.6 |

***Table S 3.*** *MIC$_{90}$ values of a selection of AAPs against type strains of the Mycobacterium avium complex. Cell shading from green to red indicates high to low activity. The displayed values are average values of two technical replicates. For detailed information on the origin of clinical isolates see Materials & Methods section of the main manuscript.*

| | *M. avium* subsp. ***hominissuis*** MAC109 | *M. avium* subsp. ***hominissuis*** M. avium 11 | *M. intracellulare* subsp. ***intracellulare*** ATCC 13950 | *M. intracellulare* subsp. ***chimaera*** CCUG 50989 |
|---|---|---|---|---|
| ID | MIC$_{90}$ [µM] | MIC$_{90}$ [µM] | MIC$_{90}$ [µM] | MIC$_{90}$ [µM] |
| **CLR** | 1.7 | 0.4 | 0.6 | 0.5 |
| **MMV** | > 50 [a] | 3.2 | 3.2 | 2.0 |
| **1** | 19.0 | 1.7 | 3.4 | 1.4 |
| **2** | 14.1 | 1.6 | 1.9 | 1.3 |
| **3** | 13.5 | 1.4 | 1.2 | 0.8 |
| **4** | 14.4 | 1.4 | 1.0 | 0.7 |
| **5** | 8.5 | 1.2 | 0.9 | 0.7 |
| **6** | 9.6 | 1.2 | 1.4 | 0.7 |
| **7** | 12.5 | 1.0 | 1.2 | 0.6 |
| **8** | 6.3 | 0.6 | 0.5 | 0.3 |
| **9** | 22.6 | 1.5 | 1.7 | 1.1 |
| **10** | 9.6 | 1.0 | 1.1 | 0.6 |

[a] incubation of *M. avium* subsp. *hominissuis* MAC109 with MMV did not reach 90% growth inhibition. MIC$_{75}$ = 6.6 µM.

***Table S 4.*** *MIC$_{90}$ values of a selection of AAPs against type strains of the different NTM. Cell shading from green to red indicates high to low activity. The displayed values are average values of two technical replicates. For detailed information on the origin of clinical isolates see Materials & Methods section of the main manuscript.*

| | *M.* ***chelonae*** ATCC 35752 | *M.* ***fortuitum*** ATCC 6841 | *M.* ***szulgai*** ATCC 35799 | *M.* ***xenopi*** ATCC 19250 | *M.* ***ulcerans*** S4018 | *M.* ***marinum*** ATCC 927 | *M.* ***simiae*** [a] ATCC 25275 | *M.* ***malmoense*** [a] ATCC 29571 | *M.* ***kansasii*** ATCC 12478 |
|---|---|---|---|---|---|---|---|---|---|
| ID | MIC$_{90}$ [µM] | MIC$_{90}$ [µM] | MIC$_{90}$ [µM] | MIC$_{90}$ [µM] | MIC$_{90}$ [µM] | MIC$_{90}$ [µM] | MIC$_{90}$ [µM] | MIC$_{90}$ [µM] | MIC$_{90}$ [µM] |
| **CLR** | 0.1 | 2.9 | 0.2 | 0.1 | 0.12 | 9.1 | > 100 | 3.4 | 0.4 |
| **MMV** | 0.8 | 1.5 | 1.6 | 48.8 | 0.47 | 2.2 | > 100 | > 100 | 0.5 |
| **1** | 0.4 | 2.0 | 2.8 | 24.1 | 0.16 | 9.6 | > 100 | > 100 | 2.6 |
| **2** | 0.3 | 0.9 | 1.4 | 21.2 | 0.09 | 3.2 | 43.0 | > 100 | 0.6 |
| **3** | 0.2 | 1.5 | 1.1 | 6.9 | 0.08 | 2.7 | 54.1 | 16.1 | 0.7 |
| **4** | 0.4 | 1.7 | 1.8 | 7.0 | 0.13 | 6.5 | > 100 | 18.2 | 0.7 |
| **5** | 0.4 | 1.4 | 0.9 | 3.3 | 0.10 | 2.4 | 45.7 | > 100 | 0.3 |
| **6** | 0.5 | 1.5 | 9.0 | 1.8 | 0.05 | 13.9 | > 100 | > 100 | 2.8 |
| **7** | 0.2 | 0.8 | 0.5 | 8.0 | 0.06 | 1.2 | > 100 | 2.3 | 0.2 |
| **8** | 0.2 | 0.6 | 0.5 | 3.3 | 0.02 | 1.4 | 20.9 | 4.9 | 0.2 |
| **9** | 0.5 | 1.6 | 1.2 | 10.0 | 0.14 | 3.1 | > 100 | > 100 | 0.8 |
| **10** | 0.4 | 1.0 | 0.7 | 5.2 | 0.08 | 1.9 | 33.5 | 5.8 | 0.5 |

[a] In some cases 90% growth inhibition was not reached for *M. simiae* ATCC 25275 and for *M. malmoense* ATCC 29571. MIC$_{75}$ values are displayed:

| | *M.* ***simiae*** ATCC 25275 | *M.* ***malmoense*** ATCC 29571 |
|---|---|---|
| ID | $MIC_{75}$ [µM] | $MIC_{75}$ [µM] |
| **CLR** | 29.3 | 0.6 |
| **MMV** | 25.2 | 3.1 |
| **1** | 36.9 | 2.8 |
| **2** | 13.7 | 1.7 |
| **3** | 16.4 | 1.8 |
| **4** | 17.9 | 2.2 |
| **5** | 11.0 | 1.7 |
| **6** | 20.9 | 5.5 |
| **7** | 6.3 | 1.1 |
| **8** | 6.9 | 0.6 |
| **9** | 20.5 | 1.7 |
| **10** | 11.2 | 1.3 |

***Table S 5.*** *Alignment of RpoB primary sequences of all tested strains from position 450-600. Position numbering refers to PDB: 5UHE. Dots represent amino acid identity.*

| | RpoB *M. **tuberculosis*** ATCC 25618 /H37Rv PDB: 5UHE | Direct drug-target contacts | Reported resistance after mutation | 7 Å distance from target bound D-AAP1 | | *M. **chelonae*** ATCC 35752 | *M. **fortuitum*** ATCC 6841 | *M. **szulgai*** ATCC 35799 | *M. **xenopi*** ATCC 19250 | *M. **ulcerans*** S4018 | *M. **marinum*** ATCC 927 | *M. **simiae*** ATCC 25275 | *M. **malmoense*** ATCC 29571 | *M. **kansasii*** ATCC 12478 | *M. intracellulare* ssp. ***chimaera*** CCUG 50989 | *M. avium* ssp. ***hominissuis*** MAC109 | *M. abscessus* ssp. ***abscessus*** ATCC 19977 | *M. intracellulare* ssp. ***intracellulare*** ATCC 13950 | *E. **coli*** ATCC 11775 |
|---|---|---|---|---|---|---|---|---|---|---|---|---|---|---|---|---|---|---|---|
| 1 | 450 | | | | T | . | . | . | . | . | . | . | . | . | . | . | . | . | . |
| 2 | 451 | | | | H | . | . | . | . | . | . | . | . | . | . | . | . | . | . |
| 3 | 452 | | | | K | . | . | . | . | . | . | . | . | . | . | . | . | . | . |
| 4 | 453 | | | | R | . | . | . | . | . | . | . | . | . | . | . | . | . | . |
| 5 | 454 | | | | R | . | . | . | . | . | . | . | . | . | . | . | . | . | . |
| 6 | 455 | | | | L | . | . | . | . | . | . | . | . | . | . | . | . | . | I |
| 7 | 456 | | | | S | . | . | . | . | . | . | . | . | . | . | . | . | . | . |
| 8 | 457 | | | | A | . | . | . | . | . | . | . | . | . | . | . | . | . | . |
| 9 | 458 | | | | L | . | . | . | . | . | . | . | . | . | . | . | . | . | . |
| 10 | 459 | | | | G | . | . | . | . | . | . | . | . | . | . | . | . | . | . |
| 11 | 460 | | | | P | . | . | . | . | . | . | . | . | . | . | . | . | . | . |
| 12 | 461 | | | | G | . | . | . | . | . | . | . | . | . | . | . | . | . | . |
| 13 | 462 | | | | G | . | . | . | . | . | . | . | . | . | . | . | . | . | . |
| 14 | 463 | | | | L | . | . | . | . | . | . | . | . | . | . | . | . | . | . |
| 15 | 464 | | | | S | T | . | . | . | . | . | . | . | . | . | . | T | . | T |
| 16 | 465 | | | | R | . | . | . | . | . | . | . | . | . | . | . | . | . | . |
| 17 | 466 | | | | E | D | . | . | . | . | . | . | . | . | . | . | D | . | . |
| 18 | 467 | | | | R | . | . | . | . | . | . | . | . | . | . | . | . | . | . |
| 19 | 468 | | | | A | . | . | . | . | . | . | . | . | . | . | . | . | . | . |
| 20 | 469 | | | | G | . | . | . | . | . | . | . | . | . | . | . | . | . | . |
| 21 | 470 | | | | L | . | . | . | . | . | . | . | . | . | . | . | . | . | F |
| 22 | 471 | | | | E | . | . | . | . | . | . | . | . | . | . | . | . | . | . |
| 23 | 472 | | | | V | . | . | . | . | . | . | . | . | . | . | . | . | . | . |
| 24 | 473 | | | | R | . | . | . | . | . | . | . | . | . | . | . | . | . | . |
| 25 | 474 | | | | D | . | . | . | . | . | . | . | . | . | . | . | . | . | . |
| 26 | 475 | | | | V | . | . | . | . | . | . | . | . | . | . | . | . | . | . |
| 27 | 476 | | | | H | . | . | . | . | . | . | . | . | . | . | . | . | . | . |
| 28 | 477 | | | | P | . | S | . | S | . | . | . | . | . | . | . | . | . | . |
| 29 | 478 | | | | S | . | . | . | . | . | . | . | . | . | . | . | . | . | T |
| 30 | 479 | | | | H | . | . | . | . | . | . | . | . | . | . | . | . | . | . |
| 31 | 480 | | | | Y | . | . | . | . | . | . | . | . | . | . | . | . | . | . |
| 32 | 481 | | | | G | . | . | . | . | . | . | . | . | . | . | . | . | . | . |
| 33 | 482 | | | | R | . | . | . | . | . | . | . | . | . | . | . | . | . | . |
| 34 | 483 | | | | M | . | . | . | . | . | . | . | . | . | . | . | . | . | V |
| 35 | 484 | | | | C | . | . | . | . | . | . | . | . | . | . | . | . | . | . |
| 36 | 485 | | | | P | . | . | . | . | . | . | . | . | . | . | . | . | . | . |
| 37 | 486 | | | | I | . | . | . | . | . | . | . | . | . | . | . | . | . | . |
| 38 | 487 | | | | E | . | . | . | . | . | . | . | . | . | . | . | . | . | . |
| 39 | 488 | | | | T | . | . | . | . | . | . | . | . | . | . | . | . | . | . |
| 40 | 489 | | | | P | . | . | . | . | . | . | . | . | . | . | . | . | . | . |
| 41 | 490 | | | | E | . | . | . | . | . | . | . | . | . | . | . | . | . | . |
| 42 | 491 | | | | G | . | . | . | . | . | . | . | . | . | . | . | . | . | . |
| 43 | 492 | | | | P | . | . | . | . | . | . | . | . | . | . | . | . | . | . |
| 44 | 493 | | | | N | . | . | . | . | . | . | . | . | . | . | . | . | . | . |
| 45 | 494 | | | | I | . | . | . | . | . | . | . | . | . | . | . | . | . | . |
| 46 | 495 | | | | G | . | . | . | . | . | . | . | . | . | . | . | . | . | . |
| 47 | 496 | | | | L | . | . | . | . | . | . | . | . | . | . | . | . | . | . |
| 48 | 497 | | | | I | . | . | . | . | . | . | . | . | . | . | . | . | . | . |
| 49 | 498 | | | | G | . | . | . | . | . | . | . | . | . | . | . | . | . | N |
| 50 | 499 | | | | S | . | . | . | . | . | . | . | . | . | . | . | . | . | . |
| 51 | 500 | | | | L | . | . | . | . | . | . | . | . | . | . | . | . | . | . |
| 52 | 501 | | | | S | . | . | . | . | . | . | . | . | . | . | . | . | . | . |
| 53 | 502 | | | | V | . | . | . | . | . | . | . | . | . | . | . | . | . | . |
| 54 | 503 | | | | Y | . | . | . | . | . | . | . | . | . | . | . | . | . | . |
| 55 | 504 | | | | A | . | . | . | . | . | . | . | . | . | . | . | . | . | . |
| 56 | 505 | | | | R | . | . | . | . | . | . | . | . | . | . | . | . | . | Q |
| 57 | 506 | | | | V | . | . | . | . | . | . | . | . | . | . | . | . | . | T |
| 58 | 507 | | | | N | . | . | . | . | . | . | . | . | . | . | . | . | . | . |
| 59 | 508 | | | | P | . | . | . | . | . | . | . | . | . | . | . | . | . | E |
| 60 | 509 | | | | F | . | . | . | Y | . | . | . | . | . | . | . | . | . | Y |
| 61 | 510 | | | | G | . | . | . | . | . | . | . | . | . | . | . | . | . | . |
| 62 | 511 | | | | F | . | . | . | . | . | . | . | . | . | . | . | . | . | . |
| 63 | 512 | | | | I | . | . | . | . | . | . | . | . | . | . | . | . | . | L |
| 64 | 513 | | | | E | . | . | . | . | . | . | . | . | . | . | . | . | . | . |
| 65 | 514 | | | | T | . | . | . | . | . | . | . | . | . | . | . | . | . | . |
| 66 | 515 | | | | P | . | . | . | . | . | . | . | . | . | . | . | . | . | . |
| 67 | 516 | | | | Y | . | . | . | . | . | . | . | . | . | . | . | . | . | . |
| 68 | 517 | | | | R | . | . | . | . | . | . | . | . | . | . | . | . | . | . |
| 69 | 518 | | | | K | . | . | R | . | . | . | . | . | . | . | . | . | . | . |
| 70 | 519 | | | | V | . | . | . | . | . | . | . | . | . | . | . | . | . | . |
| 71 | 520 | | | | V | S | . | . | . | . | . | . | . | I | . | . | S | . | T |
| 72 | 521 | | | | D | E | . | S | N | . | . | . | . | . | . | . | . | . | . |
| 73 | 522 | | | | G | . | . | . | . | . | . | . | . | . | . | . | . | . | . |
| 74 | 523 | | | | V | . | . | . | . | . | . | . | . | L | . | . | . | . | . |
| 75 | 524 | | | | V | . | . | . | . | . | . | . | . | . | . | . | . | . | . |
| 76 | 525 | | | | S | T | T | T | T | . | . | T | T | T | T | T | T | T | T |
| 77 | 526 | | | | D | . | . | . | . | . | . | . | . | . | . | . | . | . | . |
| 78 | 527 | | | | E | . | Q | . | . | . | . | . | . | . | . | . | D | . | . |
| 79 | 528 | | | | I | . | . | . | . | . | . | . | . | . | . | . | . | . | . |
| 80 | 529 | | | | V | H | D | H | . | H | H | E | . | H | H | H | H | H | H |
| 81 | 530 | | | | Y | . | . | . | . | . | . | . | . | . | . | . | . | . | . |
| 82 | 531 | | | | L | . | . | . | . | . | . | . | . | . | . | . | . | . | . |
| 83 | 532 | | | | T | . | . | . | . | . | . | . | . | . | . | . | . | . | S |
| 84 | 533 | | | | A | . | . | . | . | . | . | . | . | . | . | . | . | . | . |
| 85 | 534 | | | | D | . | . | . | . | . | . | . | . | . | . | . | . | . | I |
| 86 | 535 | | | | E | . | . | . | . | . | . | . | . | . | . | . | . | . | . |
| 87 | 536 | | | | E | . | . | . | . | . | . | . | . | . | . | . | . | . | . |
| 88 | 537 | | | | D | . | . | . | . | . | . | . | . | . | . | . | . | . | G |
| 89 | 538 | | | | R | . | . | . | . | . | . | . | . | . | . | . | . | . | N |
| 90 | 539 | | | | H | . | . | . | . | . | . | . | . | . | . | . | . | . | Y |
| 91 | 540 | | | | V | . | . | . | . | . | . | . | . | . | . | . | . | . | . |
| 92 | 541 | | | | V | . | . | . | . | . | . | . | . | . | . | . | . | . | I |
| 93 | 542 | | | | A | . | . | . | . | . | . | . | . | . | . | . | . | . | . |
| 94 | 543 | | | | Q | . | . | . | . | . | . | . | . | . | . | . | . | . | . |
| 95 | 544 | | | | A | . | . | . | . | . | . | . | . | . | . | . | . | . | . |
| 96 | 545 | | | | N | . | . | . | . | . | . | . | . | . | . | . | . | . | . |
| 97 | 546 | | | | S | . | . | . | . | . | . | . | . | . | . | . | . | . | . |
| 98 | 547 | | | | P | . | . | . | . | . | . | . | . | . | . | . | . | . | N |
| 99 | 548 | | | | I | V | . | . | . | . | . | . | T | . | . | . | V | . | L |
| 100 | 549 | | | | D | . | . | . | . | . | . | . | R | . | . | . | . | . | . |
| 101 | 550 | | | | A | . | P | . | E | . | . | . | P | . | . | D | . | . | E |
| 102 | 551 | | | | D | . | . | . | . | Q | Q | . | N | E | K | K | N | K | E |
| 103 | 552 | | | | G | . | . | . | . | . | . | . | E | . | . | . | . | . | . |
| 104 | 553 | | | | R | . | . | . | . | . | . | . | A | . | . | . | . | . | H |
| 105 | x | | | | x | x | x | x | x | x | x | x | G | x | x | x | x | x | x |
| 106 | x | | | | x | x | x | x | x | x | x | x | A | x | x | x | x | x | x |
| 107 | x | | | | x | x | x | x | x | x | x | x | E | x | x | x | x | x | x |
| 108 | x | | | | x | x | x | x | x | x | x | x | V | x | x | x | x | x | x |
| 109 | 554 | | | | F | . | . | . | . | . | . | . | . | . | . | . | . | . | . |
| 110 | 555 | | | | V | T | T | . | T | . | . | E | E | . | E | A | T | E | . |
| 111 | 556 | | | | E | . | . | . | . | . | . | . | . | . | . | . | . | . | . |
| 112 | 557 | | | | P | D | D | G | . | . | . | S | G | . | S | A | E | S | D |
| 113 | 558 | | | | R | K | . | . | . | . | . | . | . | . | . | . | K | . | L |
| 114 | 559 | | | | V | I | . | . | . | . | . | . | . | . | . | . | I | . | . |
| 115 | 560 | | | | L | . | M | . | . | . | . | . | . | . | . | . | . | . | T |
| 116 | 561 | | | | V | . | . | . | . | . | . | . | . | . | . | . | . | . | C |
| 117 | 562 | | | | R | . | . | . | . | . | . | . | . | . | . | . | . | . | . |
| 118 | 563 | | | | R | . | . | . | . | . | . | . | . | . | . | . | . | . | S |
| 119 | 564 | | | | K | . | . | . | . | . | . | . | . | . | . | . | . | . | . |
| 120 | 565 | | | | A | G | G | . | G | . | . | . | . | . | . | . | G | . | x |
| 121 | 566 | | | | G | . | . | . | . | . | . | . | . | . | . | . | . | . | . |
| 122 | 567 | | | | E | . | . | . | . | . | . | . | . | . | . | . | . | . | . |
| 123 | 568 | | | | V | . | . | . | . | . | . | . | . | . | . | . | . | . | S |
| 124 | 569 | | | | E | . | . | . | . | . | . | . | . | . | . | . | . | . | S |
| 125 | 570 | | | | Y | F | N | . | . | . | . | . | . | . | . | . | F | . | L |
| 126 | 571 | | | | V | . | . | . | . | . | . | . | . | . | . | . | . | . | F |
| 127 | 572 | | | | P | S | A | . | S | . | . | . | . | A | . | . | S | . | S |
| 128 | 573 | | | | S | A | P | . | . | . | . | . | . | . | . | . | A | . | R |
| 129 | 574 | | | | S | T | . | . | . | . | . | . | . | . | . | . | T | . | D |
| 130 | 575 | | | | E | . | D | . | . | . | . | . | . | . | . | . | . | . | Q |
| 131 | 576 | | | | V | . | . | . | . | . | . | . | . | . | . | . | . | . | . |
| 132 | 577 | | | | D | . | . | . | . | . | . | . | . | . | . | . | . | . | . |
| 133 | 578 | | | | Y | . | . | . | . | . | . | . | . | . | . | . | . | . | . |
| 134 | 579 | | | | M | . | . | . | . | . | . | . | . | . | . | . | . | . | . |
| 135 | 580 | | | | D | . | . | . | . | . | . | . | . | . | . | . | . | . | . |
| 136 | 581 | | | | V | . | . | . | . | . | . | . | . | . | . | . | . | . | . |
| 137 | 582 | | | | S | . | . | . | . | . | . | . | . | . | . | . | . | . | . |
| 138 | 583 | | | | P | . | . | . | . | . | . | . | . | . | . | . | . | . | T |
| 139 | 584 | | | | R | . | . | . | . | . | . | . | . | . | . | . | . | . | Q |
| 140 | 585 | | | | Q | . | . | . | . | . | . | . | . | . | . | . | . | . | . |
| 141 | 586 | | | | M | . | . | . | . | . | . | . | . | . | . | . | . | . | V |
| 142 | 587 | | | | V | . | . | . | . | . | . | . | . | . | . | . | . | . | . |
| 143 | 588 | | | | S | . | . | . | . | . | . | . | . | . | . | . | . | . | . |
| 144 | 589 | | | | V | . | . | . | . | . | . | . | . | . | . | . | . | . | . |
| 145 | 590 | | | | A | . | . | . | . | . | . | . | . | . | . | . | . | . | G |
| 146 | 591 | | | | T | . | . | . | . | . | . | . | . | . | . | . | . | . | A |
| 147 | 592 | | | | A | . | . | . | . | . | . | . | . | . | . | . | . | . | S |
| 148 | 593 | | | | M | . | . | . | . | . | . | . | . | . | . | . | . | . | L |
| 149 | 594 | | | | I | . | . | . | . | . | . | . | . | . | . | . | . | . | . |
| 150 | 595 | | | | P | . | . | . | . | . | . | . | . | . | . | . | . | . | . |
| 151 | 596 | | | | F | . | . | . | . | . | . | . | . | . | . | . | . | . | . |
| 152 | 597 | | | | L | . | . | . | . | . | . | . | . | . | . | . | . | . | . |
| 153 | 598 | | | | E | . | . | . | . | . | . | . | . | . | . | . | . | . | . |
| 154 | 599 | | | | H | . | . | . | . | . | . | . | . | . | . | . | . | . | . |
| 155 | 600 | | | | D | . | . | . | . | . | . | . | . | . | . | . | . | . | . |

***Table S 6.*** *Alignment of RpoC primary sequences of all tested strains from position 500-580. Position numbering refers to PDB: 5UHE. Dots represent amino acid identity.*

| | RpoC *M.* ***tuberculosis*** *ATCC 25618 /H37Rv PDB: 5UHE* | Direct drug-target contacts | Reported resistance after mutation | 7 Å distance from target bound D-AAP1 | | *M.* ***chelonae*** *ATCC 35752* | *M.* ***fortuitum*** *ATCC 6841* | *M.* ***szulgai*** *ATCC 35799* | *M.* ***xenopi*** *ATCC 19250* | *M.* ***ulcerans*** *S4018* | *M.* ***marinum*** *ATCC 927* | *M.* ***simiae*** *ATCC 25275* | *M.* ***malmoense*** *ATCC 29571* | *M.* ***kansasii*** *ATCC 12478* | *M. intracellulare ssp.* ***chimaera*** *CCUG 50989* | *M. avium ssp.* ***hominissuis*** *MAC109* | *M. abscessus ssp.* ***abscessus*** *ATCC 19977* | *M. intracellulare ssp.* ***intracellulare*** *ATCC 13950* | *E.* ***coli*** *ATCC 11775* |
|---|---|---|---|---|---|---|---|---|---|---|---|---|---|---|---|---|---|---|---|
| 1 | **800** | | | | I | . | . | . | . | . | . | . | . | . | . | . | T | . | Y |
| 2 | **801** | | | | T | . | . | . | . | . | . | . | . | . | . | . | L | . | M |
| 3 | **802** | | | | I | . | . | . | . | . | . | . | . | . | . | . | L | . | M |
| 4 | **803** | | | | V | . | . | . | . | . | . | . | . | . | . | . | P | . | A |
| 5 | **804** | | | | D | E | K | . | E | . | . | . | . | . | . | . | K | . | . |
| 6 | **805** | | | | S | . | . | . | . | . | . | . | . | . | . | . | . | . | . |
| 7 | **806** | | | | G | . | . | . | . | . | . | . | . | . | . | . | . | . | . |
| 8 | **807** | | | | A | . | . | . | . | . | . | . | . | . | . | . | . | . | . |
| 9 | **808** | | | | T | . | . | . | . | . | . | . | . | . | . | . | . | . | R |
| 10 | **809** | | | | G | . | . | . | . | . | . | . | . | . | . | . | . | . | . |
| 11 | **810** | | | | N | . | . | . | . | . | . | . | . | . | . | . | . | . | S |
| 12 | **811** | | | | F | . | L | . | . | . | . | . | . | . | . | . | M | . | A |
| 13 | **812** | | | | T | . | . | . | . | . | . | . | . | . | . | . | . | . | A |
| 14 | **813** | | | | Q | . | . | . | . | . | . | . | . | . | . | . | . | . | . |
| 15 | **814** | | | | T | . | . | . | . | . | . | . | . | . | . | . | V | . | I |
| 16 | **815** | | | | R | . | . | . | . | . | . | . | . | . | . | . | . | . | . |
| 17 | **816** | | | | T | . | . | . | . | . | . | . | . | . | . | . | N | . | Q |
| 18 | **817** | | | | L | . | . | . | . | . | . | . | . | . | . | . | . | . | . |
| 19 | **818** | | | | A | . | . | . | . | . | . | . | . | . | . | . | . | . | . |
| 20 | **819** | | | | G | . | . | . | . | . | . | . | . | . | . | . | . | . | . |
| 21 | **820** | | | | M | . | . | . | . | . | . | . | . | . | . | . | . | . | . |
| 22 | **821** | | | | K | . | . | . | . | . | . | . | . | . | . | . | . | . | R |
| 23 | **822** | | | | G | . | . | . | . | . | . | . | . | . | . | . | . | . | . |
| 24 | **823** | | | | L | . | . | . | . | . | . | . | . | . | . | . | . | . | . |
| 25 | **824** | | | | V | . | . | . | . | . | . | . | . | . | . | . | . | . | M |
| 26 | **825** | | | | T | . | . | . | . | . | . | . | . | . | . | . | . | . | A |
| 27 | **826** | | | | N | . | . | . | . | . | . | . | . | . | . | . | . | . | K |
| 28 | **827** | | | | P | . | . | . | . | . | . | . | . | . | . | . | . | . | . |
| 29 | **828** | | | | K | . | . | . | . | . | . | . | . | . | . | . | . | . | D |
| 30 | **829** | | | | G | . | . | . | . | . | . | . | . | . | . | . | . | . | . |
| 31 | **830** | | | | E | . | . | . | . | . | . | . | . | . | . | . | . | . | S |
| 32 | **831** | | | | F | . | . | . | . | . | . | . | . | . | . | . | Y | . | I |
| 33 | **832** | | | | I | . | . | . | . | . | . | . | . | . | . | . | . | . | . |
| 34 | **833** | | | | P | . | . | . | . | . | . | . | . | . | . | . | . | . | E |
| 35 | **834** | | | | R | . | . | . | . | . | . | . | . | . | . | . | . | . | T |
| 36 | **835** | | | | P | . | . | . | . | . | . | . | . | . | . | . | . | . | . |
| 37 | **836** | | | | V | I | I | . | I | I | I | . | . | . | . | . | I | . | I |
| 38 | **837** | | | | K | . | . | . | . | . | . | . | . | . | . | . | . | . | T |
| 39 | **838** | | | | S | . | . | . | . | . | . | . | . | . | . | . | . | . | A |
| 40 | **839** | | | | S | . | . | . | . | . | . | . | . | . | . | . | . | . | N |
| 41 | **840** | | | | F | . | . | . | . | . | . | . | . | . | . | . | . | . | . |
| 42 | **841** | | | | R | . | . | . | . | . | . | . | . | . | . | . | . | . | . |
| 43 | **842** | | | | E | . | . | . | . | . | . | . | . | . | . | . | . | . | . |
| 44 | **843** | | | | G | . | . | . | . | . | . | . | . | . | . | . | . | . | . |
| 45 | **844** | | | | L | . | . | . | . | . | . | . | . | . | . | . | . | . | . |
| 46 | **845** | | | | T | . | . | . | . | . | . | . | . | . | . | . | . | . | N |
| 47 | **846** | | | | V | . | . | . | . | . | . | . | . | . | . | . | . | . | . |
| 48 | **847** | | | | L | . | . | . | . | . | . | . | . | . | . | . | . | . | . |
| 49 | **848** | | | | E | . | . | . | . | . | . | . | . | . | . | . | . | . | Q |
| 50 | **849** | | | | Y | . | . | . | . | . | . | . | . | . | . | . | . | . | . |
| 51 | **850** | | | | F | . | . | . | . | . | . | . | . | . | . | . | . | . | . |
| 52 | **851** | | | | I | . | . | . | . | . | . | . | . | . | . | . | . | . | . |
| 53 | **852** | | | | N | . | . | . | . | . | . | . | . | . | . | . | . | . | S |
| 54 | **853** | | | | T | . | . | . | . | . | . | . | . | . | . | . | . | . | . |
| 55 | **854** | | | | H | . | . | . | . | . | . | . | . | . | . | . | . | . | . |
| 56 | **855** | | | | G | . | . | . | . | . | . | . | . | . | . | . | . | . | . |
| 57 | **856** | | | | A | . | . | . | . | . | . | . | . | . | . | . | . | . | . |
| 58 | **857** | | | | R | . | . | . | . | . | . | . | . | . | . | . | . | . | . |
| 59 | **858** | | | | K | . | . | . | . | . | . | . | . | . | . | . | . | . | . |
| 60 | **859** | | | | G | . | . | . | . | . | . | . | . | . | . | . | . | . | . |
| 61 | **860** | | | | L | . | . | . | . | . | . | . | . | . | . | . | . | . | . |
| 62 | **861** | | | | A | . | . | . | . | . | . | . | . | . | . | . | . | . | . |
| 63 | **862** | | | | D | . | . | . | . | . | . | . | . | . | . | . | . | . | . |
| 64 | **863** | | | | T | . | . | . | . | . | . | . | . | . | . | . | . | . | . |
| 65 | **864** | | | | A | . | . | . | . | . | . | . | . | . | . | . | . | . | . |
| 66 | **865** | | | | L | . | . | . | . | . | . | . | . | . | . | . | . | . | . |
| 67 | **866** | | | | R | . | . | . | . | . | . | . | . | . | . | . | . | . | K |
| 68 | **867** | | | | T | . | . | . | . | . | . | . | . | . | . | . | . | . | . |
| 69 | **868** | | | | A | . | . | . | . | . | . | . | . | . | . | . | . | . | . |
| 70 | **869** | | | | D | . | . | . | . | . | . | . | . | . | . | . | . | . | N |
| 71 | **870** | | | | S | . | . | . | . | . | . | . | . | . | . | . | . | . | . |
| 72 | **871** | | | | G | . | . | . | . | . | . | . | . | . | . | . | . | . | . |
| 73 | **872** | | | | Y | . | . | . | . | . | . | . | . | . | . | . | . | . | . |
| 74 | **873** | | | | L | . | . | . | . | . | . | . | . | . | . | . | . | . | . |
| 75 | **874** | | | | T | . | . | . | . | . | . | . | . | . | . | . | . | . | . |
| 76 | **875** | | | | R | . | . | . | . | . | . | . | . | . | . | . | . | . | . |
| 77 | **876** | | | | R | . | . | . | . | . | . | . | . | . | . | . | . | . | . |
| 78 | **877** | | | | L | . | . | . | . | . | . | . | . | . | . | . | . | . | . |
| 79 | **878** | | | | V | . | . | . | . | . | . | . | . | . | . | . | . | . | . |
| 80 | **879** | | | | D | . | . | . | . | . | . | . | . | . | . | . | . | . | . |
| 81 | **880** | | | | V | . | . | . | . | . | . | . | . | . | . | . | . | . | . |
